# Neurofibromatosis: New Clinical Challenges in the Era of COVID-19

**DOI:** 10.3390/biomedicines10050940

**Published:** 2022-04-19

**Authors:** Alessio Ardizzone, Anna Paola Capra, Michela Campolo, Alessia Filippone, Emanuela Esposito, Silvana Briuglia

**Affiliations:** 1 Department of Chemical, Biological, Pharmaceutical and Environmental Sciences, University of Messina, Viale Ferdinando Stagno D’Alcontres 31, 98166 Messina, Italy; aleardizzone@unime.it (A.A.); annapaola.capra@unime.it (A.P.C.); campolom@unime.it (M.C.); alessia.filippone@unime.it (A.F.); 2 Department of Biomedical, Dental, Morphological and Functional Imaging Sciences, University of Messina, Via Consolare Valeria 1, 98125 Messina, Italy; silvana.briuglia@unime.it

**Keywords:** rare diseases, neurofibromatosis, COVID-19, clinical care, personalized medicine, pharmacogenetics, telehealth, Telemedicine

## Abstract

Rare diseases constitute a wide range of disorders thus defined for their low prevalence. However, taken together, rare diseases impact a considerable percentage of the world population, thus representing a public healthcare problem. In particular, neurofibromatoses are autosomal-dominant genetic disorders that include type 1 neurofibromatosis (NF1), type 2 neurofibromatosis (NF2) and schwannomatosis. Each of the three types is a genetically distinct disease with an unpredictable clinical course and for which there is still no resolutive cure. Therefore, a personalized therapeutic approach directed at improving the symptomatology as well as the search for new pharmacological strategies for the management of neurofibromatosis represents a priority for positive outcomes for affected patients. The coronavirus disease 2019 (COVID-19) pandemic has severely affected health systems around the world, impacting the provision of medical care and modifying clinical surveillance along with scientific research procedures. COVID-19 significantly worsened exchanges between healthcare personnel and neurofibromatosis patients, precluding continuous clinical monitoring in specialized clinic centers. In this new scenario, our article presents, for the first time, a comprehensive literature review on the clinical challenges for neurofibromatosis clinical care and research during the COVID-19 pandemic health emergency. The review was performed through PubMed (Medline) and Google Scholar databases until December 2021.

## 1. Introduction

Rare diseases represent a conspicuous and heterogeneous group of human pathologies thus defined for their low diffusion in the population [1]. Despite their heterogeneity, rare diseases share several aspects which include: the difficulty for the patient to obtain an appropriate and rapid diagnosis [2], the rare availability of resolutive treatments [3], a clinical course similar to chronic disabling diseases [4], and the relevant individual, family and social burden [5].

For rare diseases, prevalence below a certain threshold is codified by specific legislation. In Europe, the criteria for these pathologies are based on a prevalence of fewer than 5 cases per 10,000 people [6], whereas in the USA, they are described as affecting fewer than 200,000 people [7].

While singularly rare, statistics data suggest that together these conditions affect a significant portion of the world’s population [3]. In particular, genetic rare diseases account for nearly 80% of all rare disorders [8], representing a serious health problem with a significant impact on the well-being of each patient and their families [9]. Thus, healthcare professionals must be aware of the resources that exist to support their diagnosis, management, and treatment.

In relation to this, a periodic biochemical–clinical monitoring of the patient and a personalized molecular genomic study are important steps for a genotype–phenotype correlation as well as for the implementation of an optimal therapeutic approach for each patient [10,11].

In this regard, the concept of “personalized medicine”, introduced in the late 1800s by the Canadian physician Sir William Osler, becomes more and more relevant considering “the great variability between individuals” [12]. In the modern era, this definition has evolved, also incorporating personal genetic/genomic information into a patient’s clinical assessment and family history to better direct medical management [13].

In this medical branch, pharmacogenetics constitutes an important goal in providing new clinical [14], diagnostic [15], and therapeutic possibilities [16], thus leading to significant progress for rare-diseases patients and improving their quality of life.

Relatedly, the main research areas concern the identification of the genetic basis of common diseases and the use of pharmacogenetic biomarkers to facilitate targeted and more effective drug therapy [17]. In this framework, it is clear how personalized medicine, also in the context of genetic-based rare diseases, represents a key element that is closely related to a continuous exchange between patients and specialized healthcare personnel.

The current health emergency, due to the coronavirus disease 2019 (COVID-19) pandemic, has made contact between patients and doctors difficult, also complicating access to medical care and examinations as well as hospital admissions [18]. All of this has resulted in a decrease in available medical visits, which has translated into less clinical monitoring and less willingness on the part of medical staff to modify the treatment plan [19,20].

In this new scenario, telehealth improved the provision of health services, representing an innovative tool in caring services while keeping patients and health providers safe during the COVID-19 outbreak.

Therefore, based on all the evidence presented above, this review aimed to analyze and summarize, for the first time, the impact of the COVID-19 pandemic on the clinical consequences of patients suffering from a rare genetic disease: neurofibromatosis.

For this purpose, we carried out a strategic bibliographic search of the current state of publications, which correlated COVID-19, Neurofibromatosis, and Telemedicine in the PubMed (Medline) and Google Scholar databases until December 2021.

To our knowledge, this is the first review that overlaps the clinical difficulties faced by patients with neurofibromatosis during the COVID-19 pandemic while indicating Telemedicine as a new solution for possible future challenges.

In such a new clinical picture, this pool of information could represent a resource in the post-COVID-19 era.

## 2. Search Strategy

### 2.1. Methods

The Pubmed (Medline) and Google Scholar bibliographic databases were used for the literature search. We considered only studies of high quality that correlated COVID-19 and neurofibromatosis patients as well as the use of Telemedicine, from the inception until the 31 December 2021. In the database terms related to COVID-19, neurofibromatosis, and Telemedicine were searched using the following keywords:

- **Neurofibromatosis**: Rare Disease, Rare Diseases, Neurofibromatosis, Neurofibromatoses, NF, NFs, NF1, NF2, Schwannomatosis.
- **COVID-19**: COVID-19, COVID19, COVID-19 Virus, COVID-19 Viruses, COVID-2019, SARS-CoV-2, SARS-CoV-2 Infection, Coronavirus, Coronaviruses.
- **Telemedicine**: Telemedicine, Telehealth, Telecare, Teleconsultation, e-health, Telerehabilitation, Video conference, remote support, phone calls.

### 2.2. Findings

After a careful literature search, we identified the following studies reported in Table 1. The studies are analyzed and discussed in Section 5.

## 3. Neurofibromatosis

The generic term “neurofibromatosis” includes at least three distinct disorders, referred to as type 1 neurofibromatosis (NF1), type 2 neurofibromatosis (NF2) and schwannomatosis [39].

The pathologies are a neurocutaneous syndrome, thus accompanied by cutaneous and neurological manifestations that cause various clinical disorders [40].

**Figure 1:**
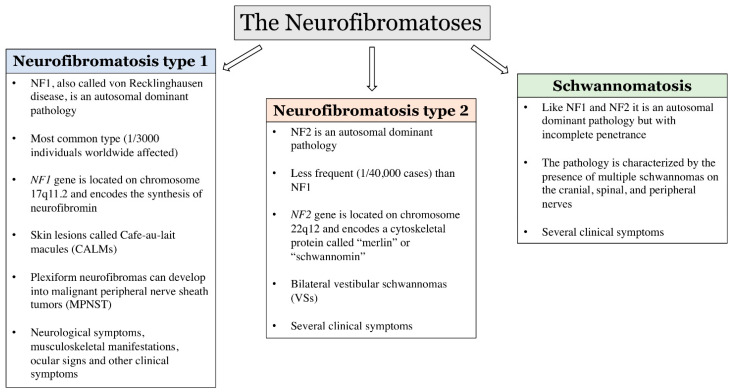
Neurofibromatoses. The figure schematizes and summarizes the main clinical and genetic characteristics of NF1, NF2 and schwannomatosis.

All three types are genetically determined dominant pathologies, but each differs from the others in a few specific characteristics, which are determined by different etiologies [41] as summarized in Figure 1.

In particular, NF1 (or von Recklinghausen disease) is the most common type [42], affecting 1/3000 individuals worldwide and occurring equally in different genders and ethnicities [43].

NF1 is caused by mutations in the NF1 tumor suppressor gene [44] and rarely by 17q11.2 microdeletions (4.2% of cases) [45].

The *NF1* gene is located on 17q11.2 region and encodes neurofibromin [46,47,48]; this protein is important in regulating Ras, a proto-oncogene that plays a prominent role in cell growth and differentiation and is mutated in many common cancers.

Neurofibromin is expressed in most tissues but at particularly high levels in the nervous system (including Schwann cells along peripheral nerve trunks, glial cells, and neurons) [49,50].

Phenotypically, genetic mutations in *NF1* manifest themselves with extremely variable clinical signs.

Although some of the patients with NF1 may remain asymptomatic, others may present with neurological symptoms [51] or bone changes [52] and especially characteristic skin lesions [53,54], which are usually evident at birth or develop during the first year of life [55].

These skin lesions consist of cafe-au-lait macules (CALMs) and freckled spots generally present on any body part, but the most common location is the trunk and the extremities [56].

In particular, cutaneous neurofibromas are one of the hallmarks of the disease, manifest in >99% of adults with NF1, and are responsible for major negative effects on quality of life [57].

These cutaneous tumors of variable size, shape and number usually arise during late childhood along the small peripheral nerves [58] and, unlike plexiform neurofibromas, are not known to have any malignant potential [59].

Plexiform neurofibromas are usually present at birth and can develop into malignant peripheral nerve sheath tumors (MPNST) over the patient’s lifetime [60,61], becoming one of the main causes of poor survival [62]. These tumor types spread along the nerve and its branches [63], causing severe pain [64] and representing the greatest vulnerability deriving from the pathology.

Moreover, NF1 patients may present neurological symptoms and disorders such as cerebrovascular disease, epilepsy, headache, and neuropathy [51]. Furthermore, NF1 can cause musculoskeletal manifestations, involving low bone mineral density (BMD), skeletal overgrowth, short stature, macrocephaly, scoliosis, skeletal dysplasia and pseudarthrosis [65,66,67,68]. On the other hand, ocular signs can also occur, including optic pathway gliomas [69] and iris hamartomas [70], also known as Lisch nodules. Other clinical symptoms comprise pulmonary hypertension [71], vasculopathy [72], and, although in low percentages, hydrocephalus [73].

NF2 has the same inheritance characteristics; it is also transmitted in an autosomal dominant way [74], but with quite different genetic and molecular features.

The NF2 gene is located on chromosome 22q12 and encodes a cytoskeletal protein called “merlin” or “schwannomin” [74,75,76]. This protein is an ezrin-radixin-moesin-(ERM)-related protein and acts as a tumor suppressor [77]; consequently, its mutation takes part in the development of tumor cells.

More specifically, the loss of merlin appears to make the cell insensitive to cellular inhibition mechanisms, typically identified as “contact-dependent growth inhibition”, which would explain tumor formation [78].

Although the pathology is much less frequent (1/40,000 cases) than NF1 [79], it usually has a very debilitating course, attributable to the presence of bilateral vestibular schwannomas (VSs) [80,81].

VSs cause progressive and disabling hearing loss, leading to social isolation and increased rates of depression in NF2 patients [82]. Despite the recourse to currently available therapies and surgical practice, NF2 still represents an unfortunate disease with a rather short life expectancy [83].

Schwannomatosis is the third major form of neurofibromatoses, clinically and genetically distinct from NF1 and NF2 [84,85]. The pathology is characterized by the presence of multiple schwannomas on the cranial, spinal, and peripheral nerves [86]. Like NF1 and NF2, it is an autosomal dominant disorder [41], but with incomplete penetrance [79], and the risk of its transmission to progeny is considerably lower than NF2 [79]. As with other forms of neurofibromatosis, there is no resolutive therapy, but treatments are aimed at managing the patient’s symptoms.

**Figure 2:**
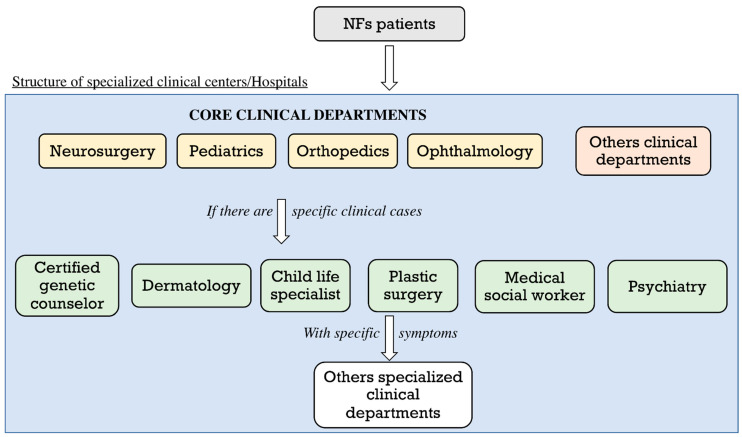
Clinical course of NFs patients. The figure illustrates the clinical path of NFs patients, also summarizing the specialist subdivision of the clinical centers.

The wide clinical spectrum expressed in these patients, at each stage in their life, underlines the importance of regular visits to qualified centers (Figure 2).

It is difficult to determine appropriate levels for patient monitoring, keeping in mind the infrequent occurrence of most complications. On the other hand, it is important to intervene with symptomatic treatments, even more effective if carried out early [43]. Age-specific monitoring of symptoms and education of patients is crucial in medical management after the diagnosis, especially in adult patients, who have to understand the signs of suspected development of MPNST. Various signs/symptoms and a high incidence of malignant tumors have a great influence on the life prognosis of patients. To raise the level of care, a multidisciplinary approach involving different departments in a dedicated clinical network in the same hospital represents the best practice to improve and promote patients’ health in order to overcome problems such as delays in medical treatment [32].

Considering the unpredictable course of these disorders, the COVID-19 pandemic did not allow this ongoing clinical monitoring, thus negatively impacting the clinical care of neurofibromatosis patients, precluding continuous medical monitoring.

## 4. COVID-19

Pandemics are major threats to life and health and require great effort to be contained and made less severe [87]. The difficulties in their management depend on many factors, starting from the unpredictability and mutability of the pathogens up to the health coordinators both at the national and international levels [87].

In this context, timely, comprehensible and as-accurate-as-possible information for both health professionals and the general population helps to maintain a high level of awareness in order to identify suspected or ascertained cases early without, however, arousing excessive alarm or intimidation [87].

In a more complete definition, an epidemic event can be considered as “the rapid spread of a particularly contagious disease in a certain more or less large area, or in a specific group of people during a definite period” [88].

This event can be caused by a new pathogen or by genetic mutations of an already pre-existing agent, which make it more virulent. An outbreak can also come from the recent introduction of an agent in an environment where it was not present before together with a different susceptibility of the host response or to new methods of contagion [87].

The SARS-CoV-2 virus was first reported in late 2019 in the city of Wuhan and has spread uncontrollably, infecting millions of people around the world [89,90].

Soon thereafter, the World Health Organization (WHO) officially declared a pandemic status [89].

Actually, according to the European Center for Disease Control, the global COVID-19 pandemic led to 435,882,971 infected patients and 5,973,364 deaths worldwide between 31 December 2019 and 27 February 2022.

Structurally, coronavirus is a spherical or pleomorphic, single-stranded, enveloped RNA virus, covered with club-shaped glycoprotein [91]. Coronaviruses are distinguished into four subtypes, as related below: alpha, beta, gamma, and delta coronavirus; each of these coronaviruses subtypes has many serotypes [91].

The virus causes acute respiratory syndrome, also generating a general malaise with flu-like symptoms [92,93]. Relatively, the most frequent clinical signs are fever, cough, headache, dyspnea, muscle and joint pain, and gastrointestinal disorders, in addition to the characteristic symptoms of COVID-19: transient loss of smell and taste [94,95,96].

The greatest risk deriving from the contraction of the virus is the onset of severe pneumonia [97], which in the most severe cases and the most vulnerable subjects can even lead to the death of the patient [98].

Although a definitive cure has not yet been established, vaccines have greatly contributed to reducing patient mortality and avoiding the most serious symptoms in the event of contagion [99]. In fact, COVID-19 vaccines have been shown to be highly effective and safe in the general population [100,101,102,103].

Firstly, four COVID-19 vaccines were authorized for use in Europe as of December 2020, listed below: the COVID-19 mRNA vaccines Comirnaty (developed by Pfizer/Biontech), Spikevax (formerly COVID-19 Vaccine Moderna), viral vector vaccines COVID-19 vaccine Janssen and Vaxzevria (formerly COVID-19 AstraZeneca vaccine) [104]. Obtaining the vaccines came quickly, thanks to a collaborative task force of regulatory agencies, pharmaceutical companies, and scientific research.

All COVID-19 vaccines authorized for administration have only obtained conditional marketing authorization. This authorization certifies that the safety, efficacy, and quality of the vaccine are demonstrated and that the benefits of the vaccine outweigh the risks while allowing developers to submit additional data on the vaccine even after marketing authorization.

Based on this, vaccine efficacy and safety will be closely monitored during the post-marketing phase, and manufacturers will need to provide more comprehensive data for further re-evaluation of the vaccine’s benefit–risk profile by regulatory agencies.

However, before and during the administration of vaccines, each country has managed the health emergency by adopting limitations aimed at containing the virus and localized outbreaks. Social distancing rules have had a drastic effect on all aspects of life, with serious socioeconomic consequences [105,106]. These negative effects of COVID-19 disease progression also had an important impact on the health system [107], including the provision of medical care [108].

All hospital medical resources have been diverted to the treatment of COVID-19 patients, thus causing the temporary suspension of routine clinical visits and care in many health centers [108].

Although the health measures adopted were fundamental to addressing the pandemic and the management of COVID patients, their impact on the health maintenance of patients in debilitating conditions caused negative outcomes for them [108,109,110] and, in particular, for neurofibromatosis patients [26].

## 5. Influence of COVID-19 Pandemic Emergency on Neurofibromatosis Clinical Features

### 5.1. Impact of the COVID-19 Pandemic on the Clinical Care of Neurofibromatosis Patients

The COVID-19 pandemic has largely affected all aspects of daily life including social relationships, economic conditions, and health care of the world population. In this health emergency, the most vulnerable subjects such as individuals with rare diseases have suffered significant inadequate care and unmet clinical needs [21].

Indeed, rare-diseases patients face a double burden of challenges due to the pandemic, addressing the uncertainty about the supply of medicines and the accessibility of essential therapies [21].

Many scientific articles [22,23] have highlighted how the pandemic has had important repercussions on health systems. Hospital centers have had to reorganize their structure to respond promptly to health emergencies [24].

From this point of view, as reported by Chowdhury et al. [21], rare-disease units around the world have also been disadvantaged. In this regard, the National Organization for Rare Disorders (NORD) of the United States recently assessed in a report the impact of the COVID-19 pandemic on patients with rare diseases [21]. In particular, 98% of patients surveyed expressed concern about the health emergency due to the pandemic. Notably, 95% of families have been directly affected by COVID-19, and over 50% have had to resort to remote appointments instead of face-to-face medical appointments. Additionally, most of the patients surveyed were worried about the potential shortage of drugs and medical supplies.

The European Organization for Rare Diseases (EURORDIS) has also reported a similar effect of the pandemic in individuals with rare diseases. In particular, 90% of European rare disease patients have experienced disruptions to routine healthcare since the start of the pandemic. Most respondents had limited access to medical therapies such as chemotherapy, infusions, and hormone treatments, or these were not done as well as other diagnostic assessments such as blood tests, heart tests, and imaging during the pandemic to limit the spread of the SARS-CoV-2 virus. This resulted in a significant loss of daily care and assistance for these fragile individuals.

More specifically, analyses conducted by the UNIAMO Italian Rare Disease Foundation and Rare Disease Ireland were in agreement with these unfavorable prospects [21]. In fact, according to the statistics reported, more than 52% of the Italian participants gave up hospital treatment to limit their exposure to possible infections [21].

On the other hand, about 46% of the participants had difficulty continuing with the prescribed therapies given the choice of the government to limit the service of health facilities to only life-saving and urgent interventions [21].

Even in the survey carried out by Rare Disease Ireland, just over 50% of the participants experienced the cancellation of scheduled medical visits [21], and in 26% of the cases, significant difficulties in accessing medicines and other medical supplies were also reported.

Similarly, Rare Disease Hong Kong (RDHK) described the interruption of medical treatments due to the pandemic in patients with 89 different rare diseases [25].

Focusing more on a specific rare disease, neurofibromatosis, it is notable that the scientific world has very little analyzed the new clinical challenges to which neurofibromatosis patients have been subjected. However, if on the one hand the insufficiency of consistent scientific data between neurofibromatosis and COVID-19 can be considered a defeat, we nonetheless hope that this examination can represent a new starting point for further future surveys.

An interesting study by Radtke et al. [26] analyzed the impact of the COVID-19 pandemic on the clinical care of neurofibromatoses. The article highlights changes in physician roles, patient volume, and medical treatment/surveillance protocols, which reduced the availability of routine neurofibromatoses-specific care. Moreover, Radtke and colleagues highlighted how the postponement of medical visits or analytical tests can delay the recognition of complications related to neurofibromatoses.

Additionally, Radtke and colleagues clarified the impact of the COVID-19 pandemic on access to new therapies for NF patients. For example, Selumetinib (Koselugo) has been approved by the FDA for the treatment of plexiform neurofibromas in pediatric patients with NF1 during the pandemic. Concerning this, the study showed that although 22% of NF clinics were ready to start treatment with suitable patients, on the other hand, 63% were waiting for an in-person appointment with patients to discuss therapy.

In addition, 12% of the clinics had postponed the initiation of a new therapy in patients under treatment until after the resolution of the pandemic crisis [26].

Analogously, Wolters et al. [27] in their online survey reported that due to the state of the COVID-19 pandemic, approximately 30% of participants had missed a face-to-face doctor’s appointment for NF care and just under 20% had missed a treatment at the hospital because it was canceled or postponed.

Almost half (48.3%) of adults who missed a doctor’s visit for NF care reported being quite concerned about the interruption of continuity of care.

Moreover, in the NF patients who missed a treatment, the majority (64.2%) reported moderate to high apprehension.

The lack of a NF-related medical appointment due to the health emergency has been associated with an increased state of concern regarding possible coronavirus infection. This condition during the pandemic had repercussions on the physical and emotional health of NF patients, increasing the state of stress related to the spread of the coronavirus.

To this, as indicated by Wolters and colleagues, is also added the inability of NF patients to undergo tumors surgery, thus causing more serious healthcare needs in the future.

Recently, two clinical case reports described NF1 patients affected by COVID-19 with infrequent medical complications. A 68-year-old woman with severe respiratory failure due to COVID-19 was treated with veno-venous extracorporeal membrane oxygenation (VV-ECMO) and had frequent bleeding complications, requiring multiple transcatheter arterial embolizations (TAE) [28]. The indication and benefits of VV-ECMO should be considered carefully for patients with NF1 and a non-anticoagulation strategy may be effective [28].

In the other case, a 60-year-old male patient, previously diagnosed with NF1, developed pneumothorax and pleural effusion during the recovery period after severe COVID-19 pneumonia [29].

The health emergency caused by the COVID-19 pandemic has also had a severe impact on clinical and health research, resulting in the suspension of several translational, clinical, and basic science investigations [111], affecting every side of medical practice.

In fact, in the last year, numerous clinical trials have been abandoned, suspended, or postponed [112,113]. Consequently, this could result in a potential reduction in drug discovery and/or management strategies for diseases other than COVID-19, such as cancer, cardiovascular disease, and even rare diseases [31]. Research related to SARS-CoV-2 has become the focus of many researchers, causing a very sudden slowdown in medical research on diseases other than COVID-19, thus making rare-disease research more challenging and slower [21].

In this regard, Radtke et al., advised that considering the insufficiency of approved treatment options for neurofibromatosis, even temporary delays in seeking clinical trials can be devastating for patients and their families [26].

Therefore, while fighting the COVID-19 pandemic through new vaccination campaigns, diagnostic and research services on rare diseases must not be overlooked but represent an absolute priority to ensure proper management of rare diseases and the safeguarding of less fortunate patients.

New insights should be adopted to improve the management of NF patients, such as the use of recombinant transgene sequences enveloped into viral vectors [114]. To standardize and raise the level of clinical care for neurofibromatosis nationwide and integrate research into clinical practices, a multidisciplinary approach should be promoted and encouraged to support patients in specialistic centers [32].

In this perspective, in addition to adequate therapeutic support, psychological support should also be provided to NF patients.

Several studies [33,34] indicated how the COVID-19 pandemic and the established public health measures had serious psychological repercussions on all individuals and even more on patients suffering from rare diseases [27,35].

Accordingly, the increase in depressive and anxious states contributes to negative outcomes on the health and psychological wellbeing of rare-diseases patients [35].

In particular, a survey conducted by Wolters and colleagues highlighted how NF patients suffered from moderate-to-high amounts of concern about the impact of COVID-19 on their emotional (46.3%) and physical health (46.7%); in addition, 54.8% reported moderate-to-high pandemic-related stress [27].

### 5.2. New Strategies to Fight the Impact of the COVID-19 Pandemic

Telemedicine aims at the use of telecommunication systems to provide remote health services like the provision of specialist consultancy, the monitoring of patients suffering from pathologies, and the facility of consultancy for the self-management of their pathology [115].

All these aspects can contribute to the improvement of the patient’s outcomes, also reducing health care costs, both public and private [116].

Considering the social distancing rules implemented following the COVID-19 health emergency, Information Technology (IT) resources could represent a valid help in providing psycho-therapeutic support to neurofibromatosis patients. In particular, Telemedicine could represent an effective solution to maintain contact between doctors and patients, guaranteeing safety.

It consists of a series of interventions, including diagnosis, evaluation, and treatment carried out through telecommunication technologies (for example, video calls), which allow the patient to perform the therapy directly at home and to have immediate and precise feedback from the clinician [115].

As demonstrated by compelling evidence [36,37], the advantages are considerable; in fact, Telemedicine allows doctors to provide medical assistance even to patients who are in remote districts, reduces health costs, and reduces the transmission of infectious diseases, thus offering promising potential in the fight against COVID-19 [38].

Despite the clinical difficulties due to the COVID-19 pandemic, Telemedicine was immediately used (within 2–3 months) by specialized neurofibromatosis clinics. Data reported by Radtke et al. [26] suggest a notable increase in Telemedicine, from only 2% of pre-pandemic clinics to 98% of clinics during the pandemic.

However, the majority (84%) of the clinics analyzed indicated that maintaining adequate insurance coverage was essential to continue using Telemedicine for NF patient care. On the other hand, the remaining clinics were unlikely to continue adopting Telemedicine. The opinion of some specialists was that of using Telemedicine only in specific cases, such as for stable or uncomplicated follow-up patients and families traveling at a distance, or unable to go to the hospital, or for needs of patients. Only in such circumstances would Telemedicine visits represent a good alternative to face-to-face visits.

A case report involving a NF 49-year-old man with complete deafness elucidated the benefits of telerehabilitation as a solution to provide rehabilitation to quarantined individuals [30]. Telerehabilitation was conducted through an Internet-based remote communication system so that patients could be provided with precise exercise instructions and medical information [30].

This strategy has proved advantageous, leading to excellent results such as a successful exercise program, thus avoiding patient functional decline, and a high degree of patient satisfaction.

Similarly, Wolters et al. [27] reported that 43.4% of respondents used telehealth to overcome COVID limitations.

Of those individuals, 33.3% stated that the telehealth appointment discreetly met their needs, whereas 46.2% stated an extremely favorable attitude toward Telemedicine [27].

This survey also indicated that patients with learning difficulties were less likely to use Telemedicine [27]. Furthermore, NF patients interviewed with moderate/severe symptoms were more likely to use Telemedicine than subjects with mild clinical signs [27].

Conversely, other factors, including age, gender, mental diseases, and education level, were not significantly related to the use of Telemedicine [27].

In addition, language differences do not represent a barrier but can be overcome by using simultaneous automatic translation devices to better understand the therapist’s instructions [30].

Likewise, EURORDIS reports that in this period most of the consultants are trying to provide support and services to people with rare diseases through technological aids such as telephone calls, videoconferencing, etc. [21].

It was reported that nearly 50% of the respondents had used the Telemedicine service, as in-person visits were not recommended [21].

These data are promising and encourage exploration of new care options that, while unable to fully replace face-to-face medical visits, ensure remote support for rare disease patients.

## 6. Limitations

Several limitations should be considered. We have tried to describe the studies in as much detail as possible, trying to elucidate two key themes: the impact of the COVID-19 pandemic on the clinical care of patients with neurofibromatosis and the use of new medical strategies to overcome the lack of medical visits. Despite this, the limitations encountered during this study are mainly due to the topics covered. Few articles deal with the challenges faced by patients with rare diseases, and in particular patients with neurofibromatosis. In addition to this, the short time frame analyzed must also be considered, as the pandemic spread just two years ago.

## 7. Conclusions and Future Perspectives

The COVID-19 pandemic strained the entire world population and in particular the health system, which has had to face new clinical challenges caused by an unprecedented pandemic. The extraordinary influx of COVID-19 patients required a high intensity of care, which overshadowed the clinical care of patients suffering from life-threatening diseases such as neurofibromatosis. However, this new health landscape, caused by the COVID-19 widespread, promoted the adoption of innovative solutions in the provision of medical assistance.

Concerning this, in the era of COVID-19, Telemedicine has represented and continues to represent a valid support in the management of patients suffering from rare diseases, including neurofibromatosis. In these promising perspectives, the use of new medical technologies could be an important step forward in verifying the patient’s adherence to therapy, in the timely measurement of the patient’s anamnestic characteristics as well as in teleconsulting, thanks to videoconferencing systems easily accessible via mobile devices.

Therefore, its effective implementation also in post-COVID digital health care would guarantee the continuous improvement of the quality of health services and the achievement of essential levels of assistance, also favoring high levels of monitoring of the health system’s performance and multidisciplinary counseling.

Moreover, genetic counseling in remote mode to guarantee the ongoing clinical genetics service was performed by video call during the pandemic—for example, to receive preconception genetic counseling to discuss inheritance risks, the range of reproductive options, and the variability of the manifestations of the diagnosed condition. By contrast, surgical procedures, prenatal genetic testing, or comprehensive genetic counseling in suspicion of NF disease require face-to-face approaches.

Future emergencies could pose a similar or even higher risk for the NF population. More information regarding other factors such as lifestyle, co-morbidities, response to treatments, and vaccination in NF subjects can be important in medical practice, considering the special needs of these higher-risk subjects.

## Figures and Tables

**Table 1:** The table summarizes the articles on neurofibromatosis, COVID-19 challenges, and Telemedicine.

First Author	Year	Type of Study	Analysis/Outcomes	References
Chowdhury	2021	Literature Review	The article analyzed the impact of COVID-19 Pandemic on rare diseases patients	[21]
Armocida	2020	Editorial	Report of Italian health system and the COVID-19 challenge	[22]
Blumenthal	2020	Literature Review	Analysis of COVID-19 and implications for the Health Care System	[23]
Talarico	2020	Literature Review	Analysis of Rare Disease and Global Health Emergency	[24]
Chung	2020	Clinical research	Impact of COVID-19 pandemic on patients with rare disease in Hong Kong	[25]
Radtke	2021	Clinical survey	Sixty-three United States NF clinics online survey	[26]
Wolters	2022	Clinical survey	Anonymous online survey distributed to adults with NF. 613 adults (18–81 years; Mean = 45.7) with NF1 (77.8%), NF2 (14.2%), and schwannomatosis (7.8%). The analysis assess the impact of the pandemic on mental health and NF health care	[27]
Shimoyama	2021	Case report	A patient with COVID-19 and bleeding complications due to neurofibromatosis type 1 during VV-ECMO.	[28]
Wakamatsu	2021	Case report	A case of a patient with neurofibromatosis type I who developed pneumothorax and eosinophilic pleural effusion after suffering from COVID-19 pneumonia	[29]
Tatemoto	2021	Case report	Successful telerehabilitation delivery for patient (49-year-old man with NF) quarantined due to COVID-19	[30]
van Koningsbruggen-Rietschel	2020	Editorial	The article analyzed how SARS-CoV-2 disrupts clinical research in the rare disease-specific trial network	[31]
Nishida	2021	Clinical article	The study analyzed the establishment of an in-hospital clinical network for patients with neurofibromatosis type 1 in Nagoya University Hospital	[32]
Le	2021	Clinical article	The study analyzed the psychological consequences of COVID-19 lockdowns	[33]
Orgilès	2020	Clinical survey	The study analyzed the psychological consequences of COVID-19 quarantines in young people	[34]
Sanchez-Garcia	2021	Online study	The study analyzed the depression and anxiety in patients with Rare Diseases during the COVID-19 Pandemic	[35]
Portnoy	2020	Editorial	The study analyzed the use of Telemedicine during the COVID-19 pandemic	[36]
Bashshur	2020	Editorial	The study analyzed the use of Telemedicine during the COVID-19 pandemic with future perspectives	[37]
Bokolo	2021	Literature Review	The study analyzed the adoption of Telemedicine and virtual software for care of outpatients during and after COVID-19 pandemic	[38]
